# Kinematically aligned total knee arthroplasty via subvastus approach using the Mako® 2.0 total knee system: a surgical technique

**DOI:** 10.3389/fsurg.2026.1653726

**Published:** 2026-03-24

**Authors:** Teddy Cheong, Jonathan Yeo, Charles Kon Kam King, Ing How Moo

**Affiliations:** Orthopaedic Surgery, Changi General Hospital, Singapore, Singapore

**Keywords:** arthroplasty, kinematic alignment, knee, Mako®, robotic

## Abstract

Total knee arthroplasty (TKA) is among the most commonly utilised and cost-effective surgical musculoskeletal procedures, with up to 700,000 TKAs performed annually in the United States alone. Traditionally, the standard approach in TKA has been for mechanical alignment (MA) which provides equal medial and lateral gaps in extension and flexion. However, there remains a high rate of patient dissatisfaction with their TKA. Kinematic alignment (KA) aims to restore pre-arthritic alignment by placing the femoral and tibial components in alignment with the three kinematic axes of the knee while avoiding soft tissue releases. KA TKAs have produced favourable clinical outcomes and implant survivorship that are comparable and even superior to mechanical alignment TKAs in some studies. As technology advances, robotic-assisted systems such as the Mako® 2.0 Total Knee system are increasing in popularity due to its benefit of surgical precision and accuracy. To perform a KA TKA effectively, accurate bone resections, particularly the femur aspect, are required to ensure that the pre-arthritic alignment and joint line are restored. The Mako® 2.0 Total Knee system provides accurate and precise assistance in performing a KA TKA through the combination of meticulous computer tomography-based pre-operative planning and AccuStop™ haptic technology. Currently, there is a lack in existing literature that describes the technique of performing a KA TKA while using Mako® Total Knee 2.0 system. In this article, we describe specific procedures for pre-operative planning, intra-operative steps, and soft tissue evaluation using the Mako® 2.0 Total Knee system to perform a KA TKA. The aim is to describe a practical and efficient workflow that integrates kinematic alignment principles with the precision, reproducibility, and intraoperative feedback provided by robotic assistance.

## Introduction

Total knee arthroplasty (TKA) is among the most commonly utilised and cost-effective surgical musculoskeletal procedures, with up to 700,000 TKAs performed annually in the United States alone (1). The utilisation rate has increased over the last three decades and is projected to increase even further with an aging population (2). Traditionally, the standard approach in TKA has been for mechanical alignment (MA), in which the femoral and tibial components are implanted perpendicular to the tibial and femoral mechanical axis, with equal medial and lateral gaps in flexion and extension achieved through ligamentous release (3). Despite technological advancements such as patient-specific instrumentation and robot-assisted surgery, achieving accurate neutral MA has not shown to significantly contribute to improvement in functional outcomes (4). In fact, residual knee symptoms such as pain, instability, and effusion have been reported in up to 50% of patients after MA TKA. There remains a high rate of patient dissatisfaction, reaching almost 20% in some reports (5–7). These residual complications suggest intrinsic technical limitations of MA TKA, owing to non-physiological prosthetic knee anatomy, balance, and kinematics (8–10). Studies have shown that natural, pre-arthritic alignment of the knees are usually not perfectly neutral (11–14). The limitations of MA have led to the development of various alignment philosophies, one of which is kinematic alignment (KA). The KA concept was originally theorised by Stephen Howell and aims to restore pre-arthritic or native constitutional alignment by placing the femoral and tibial components in alignment with the three kinematic axes and joint lines of the native knee without the need for ligamentous releases and without restrictions on the degree of preoperative varus, valgus, flexion, posterior tibial slope, and the degree of postoperative correction (15, 16). Thus, this restores the native laxities, tibial compartment forces, Q-angle, and hip-knee-ankle angle (16–18). When compared to MA, KA has shown superior clinical outcomes, whilst maintaining similar revision rates, with a 16-year implant survivorship of 93 (19–23). Recent technological innovations have led to the development of robotic-assisted systems to enhance surgical precision, such as the Mako® robot-assisted system.

The Mako® robot-assisted system for TKA is a safe and reliable procedure, associated with more precise resection, accurate component positioning and alignment when compared with conventional TKA (25–27). Studies have reported at least equal or superior functional outcomes when compared to conventional TKA (24, 27–28). Since the introduction of the Mako® system, there has been an increase in its usage throughout the world with over 1.5 million Mako® procedures performed across over 45 countries around the world according to the 2024 Mako® statistics (29).

With the advancement of technology in the field of Orthopaedics, it is important for surgeons to adapt and utilise new technologies to enhance their surgical precision and improve patient outcomes. KA TKA was initially a callipered measurement technique as described by Howell (16). To the best of our knowledge, an in-depth description of the surgical technique for KA TKA using the Mako® robot-assisted system has not been described in the literature. This article describes the surgical technique for KA TKA using the Mako® 2.0 Total Knee system. The aims are to provide specific procedures for pre-operative planning, intra-operative steps, and soft tissue evaluation using the Mako® 2.0 Total Knee system to perform a KA TKA.

## Pre-operative planning

Pre-operative planning for a robotic-assisted TKA is essential. This begins with basic knee pre-operative radiographs and a Mako® protocol computer-tomography (CT) scan of the knee joint. The CT images are used to generate detailed three-dimensional (3D) bone models of the femur and tibia, excluding the cartilage. The Mako® system provides a patient-specific three-dimensional bone model derived from preoperative CT imaging thus allowing the surgeon to visualize individual anatomy, joint line orientation, and bony morphology. Quality of the scan is assessed, and pre-operative planning is performed to identify the key landmarks of the femur and tibia and digitally plan the implants that will be used. Implant sizing, component position, and planned resections are dynamically adjusted by the surgeon based on patient-specific anatomy, desired kinematic alignment targets, and intraoperative assessment of gap balance and soft-tissue tension. Bone density does not influence the execution of the kinematic alignment strategy or the determination of planned resection thickness. Differences in bone density are managed at the level of surgical execution and fixation technique, rather than through modification of alignment or resection planning.

### Femoral resections

**Figure 1:**
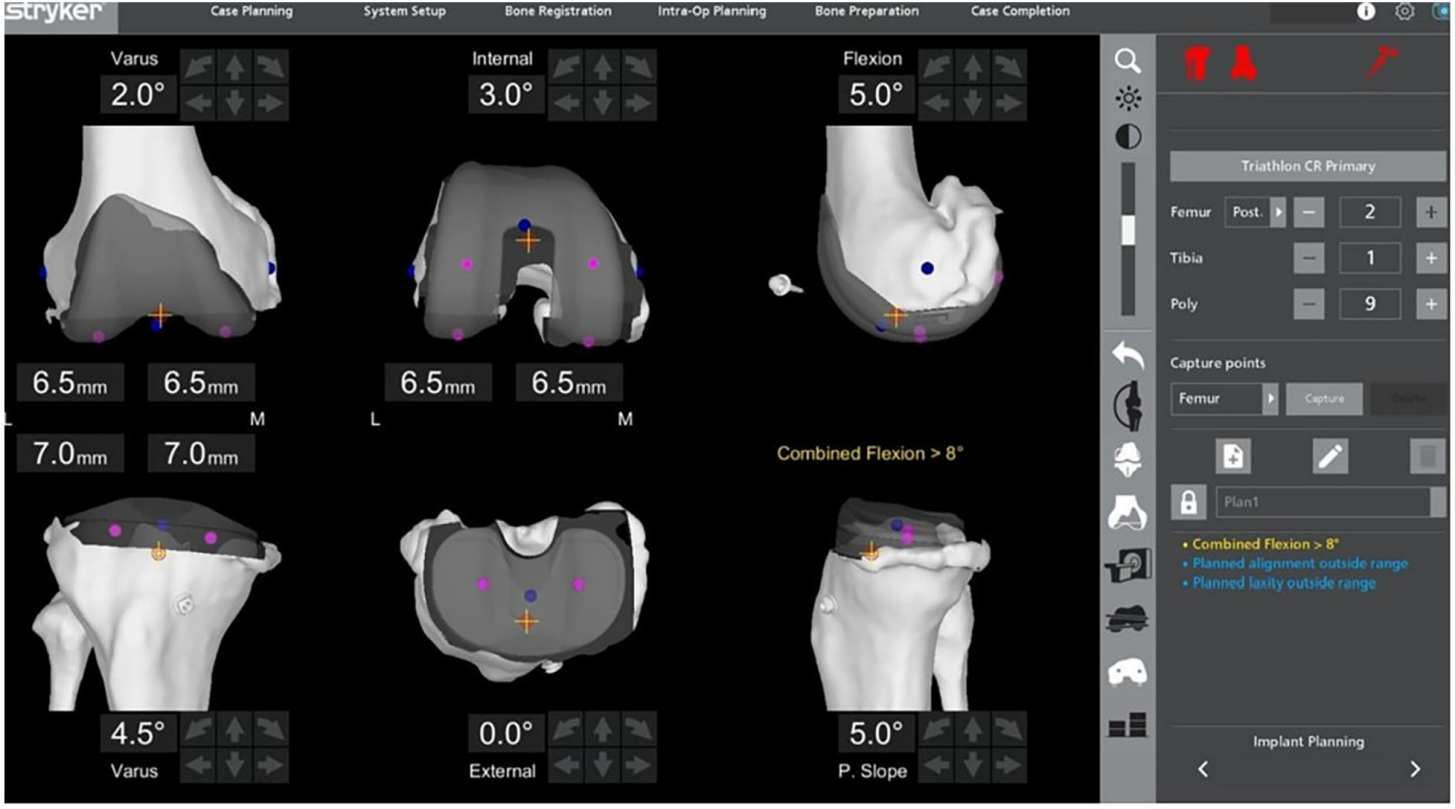
An example of pre-operative planning. Implant sizes are set based on the computer tomography scan bone dimensions. Posterior and distal femur cuts are set at 6.5 mm, while the tibia cuts are set at 7 mm medially and laterally. Valgus-varus angles displayed should be the native alignment. Posterior slope is adjusted until the tibial implant is parallel to this medial joint surface.

The distal and posterior femur bone cuts are set at 6.5 mm (Figure 1). This value is set based on two factors. Firstly, the Stryker® Triathlon femoral component has an implant thickness of 8.5 mm (30). Secondly, full-thickness cartilage loss in a human knee is approximately 2 mm (31, 32). Cartilage thickness is variable and may be influenced by factors such as age, sex, body mass index, anterior cruciate ligament status, and limb alignment. However, reported cartilage thickness in the healthy adult knee generally ranges between approximately 1.8 mm and 3.1 mm, with multiple studies demonstrating an overall mean thickness of approximately 2.0–2.3 mm across the distal and posterior femur. *In vivo* validation studies have demonstrated that cartilage thickness values used during TKA planning closely approximate these accepted averages. Johnson et al. validated articular cartilage depth intraoperatively and reported values consistent with an average thickness of approximately 2.0 mm for the distal and posterior femur (33) Similarly, magnetic resonance image (MRI) based assessments have demonstrated a mean cartilage thickness of approximately 2.28 mm across knees (34). As the cartilage loss amount is not visualised or quantified in the Mako® pre-operative planning bone model, the planned bone resection is derived from subtracting the cartilage loss (2 mm) from the femoral implant thickness (8.5 mm) which results in 6.5 mm cut. This amount of femur cut is the standard in all KA TKAs done with the Mako® 2.0 system by the senior surgeon and is easily reproducible. The femoral resection amount remains unchanged, in accordance with the foundational principle of kinematically aligned TKA as a femur-based, purely mechanical resection technique that aims to restore the native joint by simply resurfacing the bone without altering its pre-arthritic anatomy (15, 16). Previous literature on non-robotic KA TKA has shown that the use of manual instrumentation can reliably position the femoral component in all three anatomical planes—coronal, sagittal and axial (18). With the aid of the Mako® 2.0 system, femoral planning and component placement are further streamlined, improving both accuracy and reproducibility. In term of sagittal alignment for femoral component, the component was flexed between 0° and 9° as necessary to achieve the best fit. Best fit in this context is defined by a combination of objective criteria. Firstly, avoidance of anterior femoral notching which is achieved by avoiding excessive component extension and accounting for the native sagittal femoral bow. This also helps prevent patellofemoral overstuffing. Secondly, avoidance of excessive femoral component flexion, which can reduce the anteroposterior dimension of the distal femur and result in unintended femoral component under sizing. Lastly, maintenance of the planned resection depth to preserve restoration of the native joint line and joint height in accordance with kinematic alignment principles.

This surgical planning and its associated adjustments naturally establish the proximal-distal position and varus-valgus orientation of the femoral component to coincide with the native joint line, while also aligning the anterior-posterior position and internal-external rotation of the component with the native posterior condylar axis (PCA). The femoral mechanical axis, trans-epicondylar axis (TEA), and Whiteside's line are not of interest or use when kinematically aligning the femoral component.

### Tibia resections

The next step is to adjust the tibia bone cuts. The amount of tibia resection is derived in a similar fashion to the femur component. In contrast to the traditional TKA, the reference point for tibial cut in KA is referenced off from the base of the tibial spines. The Stryker® Triathlon tibia component has a minimum thickness of 9 mm (30). Thus, the tibia resection is set to 7 mm as the cartilage is not being accounted for in the CT bone model scan (Figure 1). The posterior slope of the tibial resection is referenced from the native medial tibial joint line in the sagittal plane. It is adjusted until the tibial implant is parallel to this medial joint surface, ensuring alignment with the native anatomy. This surgical planning will have achieved a proper proximal–distal position, as well as the varus–valgus, flexion–extension, and internal–external orientations of the tibial component, that are aligned with the native tibial joint line (Figure 1). In KA TKA, the tibial mechanical axis, intramedullary canal, and tibial tubercle are not utilized or considered relevant, as they do not contribute to restoring the native joint anatomy.

It is important to note that the calliper-verified resections for both the femur and tibia will measure 2 mm less than the resection values set in the Mako® pre-operative planning system. This is due to the saw blade kerf thickness being 2 mm. The planned resection values stated above represent the intended amount of bone to be removed by the surgeon and therefore inherently account for the saw blade kerf. As such, no additional numerical adjustment for kerf is required at the planning stage. This will be explained further in the “Performing and Validating the Bone Cuts” section.

### Varus/valgus angles and posterior slope

At this point, the native valgus/varus angle of the femur and tibia is restored. Thus, these values reflected are not adjusted further. This differs from the concept of restricted Kinematic Alignment (rKA) principles proposed by Vendittoli et al., where the amount of accepted valgus-varus angle is restricted (35). Howell et al. prospectively reviewed 214 knees and found that KA TKA restores function without catastrophic failure regardless of the degree of valgus-varus alignment, even those that were categorised as “outliers” (36). In a further review by Howell et al., on 16-year outcomes of 222 KA TKAs, it was reported that not restricting the pre-operative deformity did not adversely affect the 16-year implant survival, yearly revision rate and function level (23).

In KA, femoral component rotation is determined by restoration of the patient's native, constitutional anatomy rather than by applying a fixed external rotation (e.g., 3°) relative to the PCA as is commonly performed in mechanical alignment. Femoral rotation is therefore set at 0° with respect to the patient's posterior condylar axis, placing the femoral component parallel to the native joint line rather than externally rotating it to an arbitrary target (15). This approach preserves native joint line orientation and femoral kinematics. As mentioned, the TEA is ignored as it is not relevant in the principle of KA (15) (Figure 1).

## Surgical set-up

A standard operating theatre set-up for a TKA is adopted with the patient prepared in a supine position. The surgeon is positioned on the side of the operated leg, with the Mako® robotic arm entering from the same side. The display monitor and camera stand are placed on the opposite side. As a direct-line-of-sight is required for all tracking arrays, the non-operative side must be free of any obstruction to the camera during the steps of the procedure that require real-time tracking.

## Skin incision and exposure

No tourniquet is used during the procedure. A standard midline skin incision is performed, and a sub-vastus approach is adopted by the senior author. The use of the Mako® Total Knee System reduces the need for direct visualization, allowing for a smaller incision and less extensive soft tissue dissection during bone preparation (37, 38). This minimally invasive approach may contribute to reduced postoperative pain and faster postoperative rehabilitation.

## Placement of arrays and landmarking

**Figure 2:**
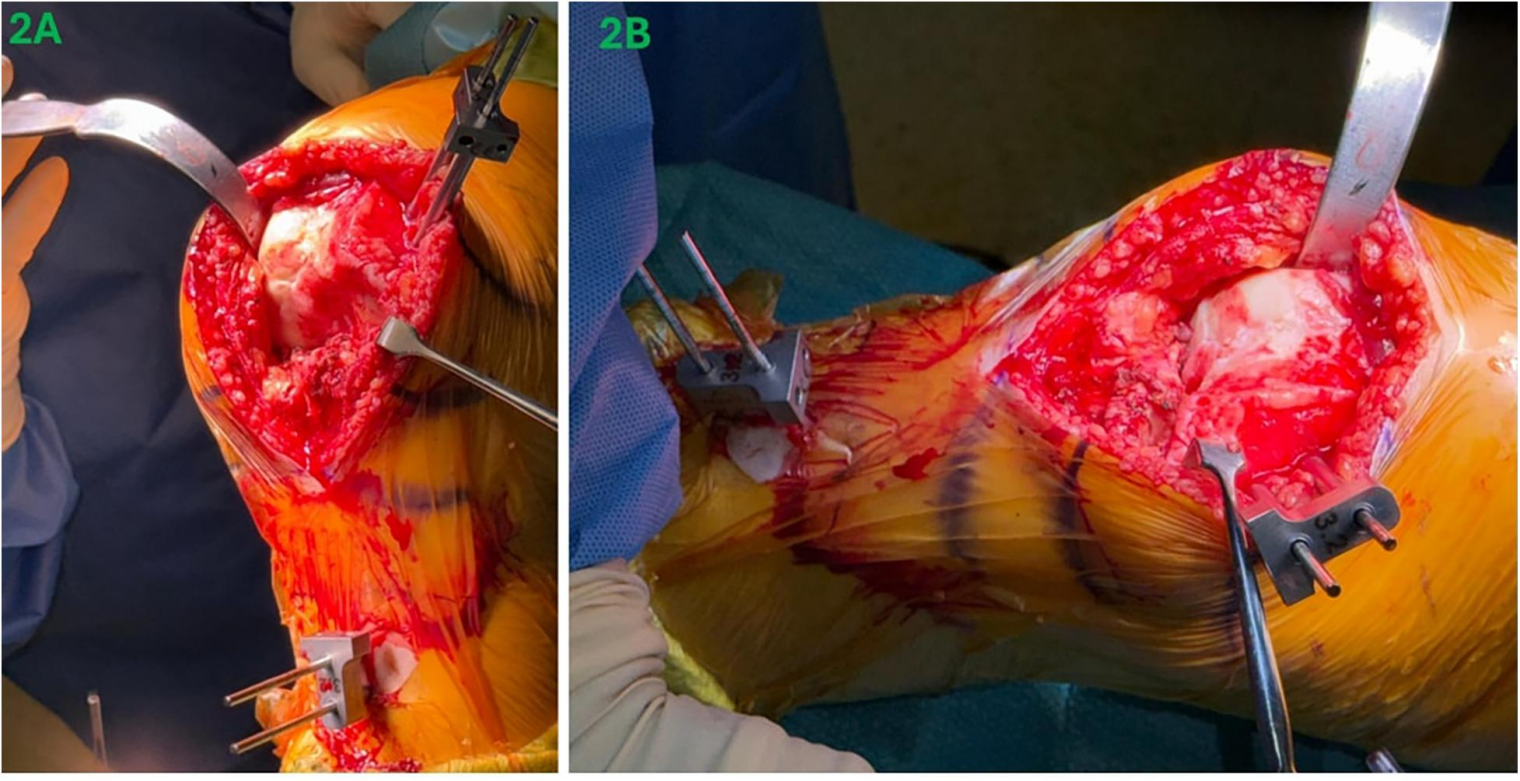
Femur pins **(A)** are placed “in wound” through the incision. Tibia pins **(B)** are placed inferior to tibia tubercle and medial to the tibial crest.

Femoral and tibial arrays are then placed using 3.2 mm threaded pins. The senior author prefers to place the femur pins “in wound” through the midline incision to prevent additional skin incisions with the knee flexed to 90°. The femoral pin will also serve as a medial retractor during the procedure, enhancing exposure while minimizing soft tissue trauma. The femoral pins are inserted approximately 1 cm anterior and proximal to the origin of the medial collateral ligament (MCL). They are aimed parallel to the joint line in the coronal plane and at a 45° angle in the axial plane to avoid interference with the surgical field (Figure 2). Also, ensure that the barrels of the array stabiliser are directly on the bone surfaces to ensure stability. A stab incision is made approximately 10 cm inferior to the tibia tubercle and 1 cm medial to the tibial crest. The first tibial pin is drilled through this incision, followed by the proximal sleeve of the array stabiliser placed through this pin. The second pin location is determined through the distal sleeve of the array stabiliser (Figure 2). Landmarking is performed according to the Mako® TKA Surgical Guide (39). The authors recommend keeping the error of registration to less than 0.2 mm. In our workflow, a registration error of less than 0.2 mm was chosen as a practical threshold to ensure reliable reproduction of the patient's native joint anatomy during kinematic alignment. If this accuracy is not achieved, the implications include potential deviation from intended kinematic alignment targets, which may affect soft-tissue balance and gap symmetry. When the registration error exceeds 0.2 mm, the decision is made not to proceed with bone resections. Instead, systematic troubleshooting is performed, including reassessment of bony landmark identification, and repeat surface mapping when necessary.

## Initial evaluation

**Figure 3:**
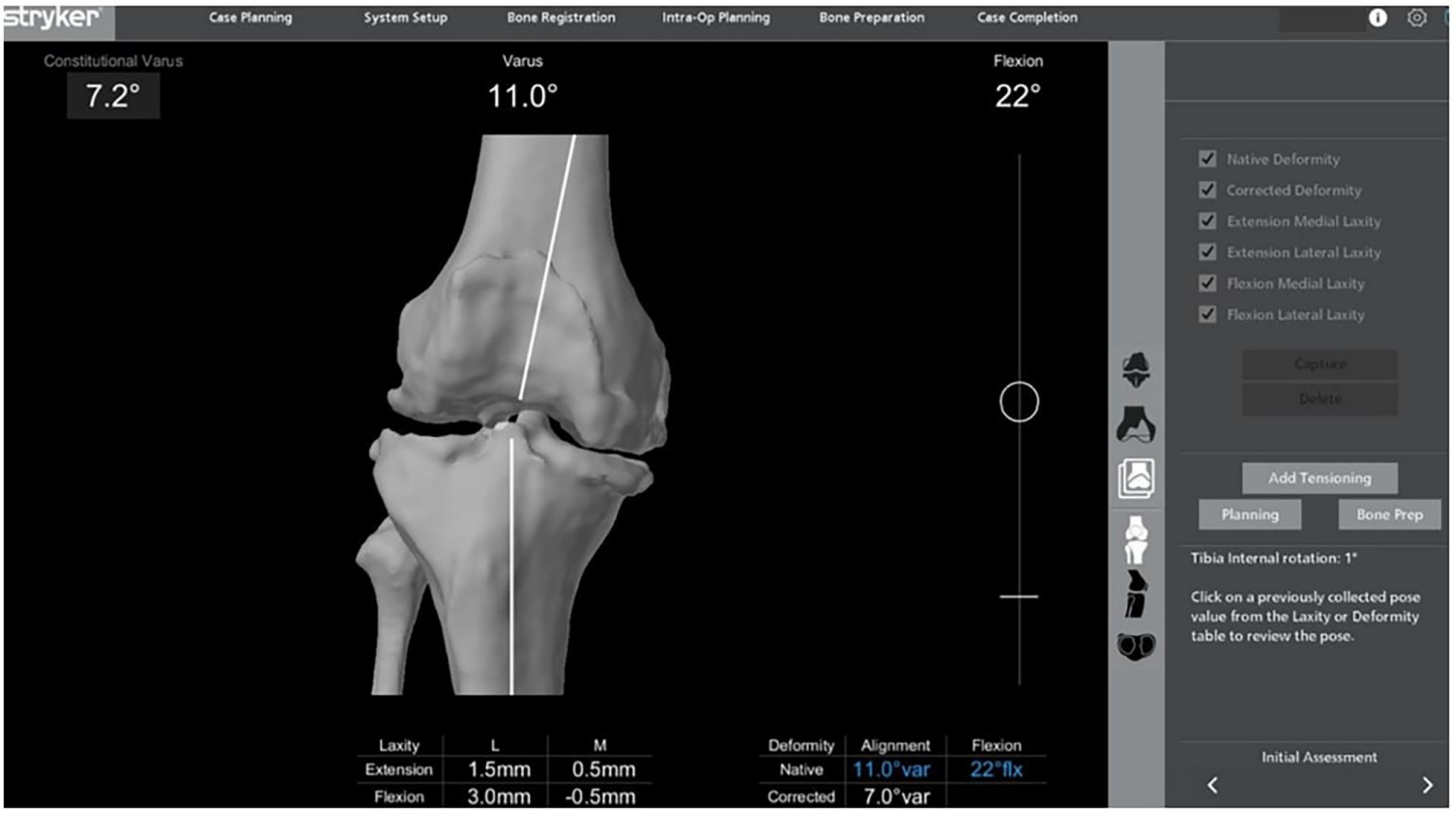
An example of the initial evaluation. The native alignment is 11° varus, and the aim is to achieve 7° varus alignment after corrective stresses are applied. The medial and lateral laxities in both extension and flexion are displayed and the medial compartment is found to be tighter especially in flexion.

After removal of the accessible osteophytes, the soft tissue balance was evaluated with the planned position of the component by exerting varus and valgus stresses to obtain maximal gaps. As part of the Mako® workflow, gap measurements were performed at 20° extension and 90° flexion while varus and valgus stress were applied to the knee. Laxity testing occurs at about 20° of flexion in the Mako® system compared to the conventional KA technique where laxity is checked in 0° of knee flexion (Figure 3). It is also important to note that the physiological extension and flexion gaps are asymmetric (40). Even with the knee in 20° of flexion, there is asymmetry in the gaps between the medial and lateral side. The lateral compartment is generally looser, especially in flexion (41, 42). Compared to the medial compartment, the lateral compartment has 0–2 mm more laxity in extension and 1–4 mm more laxity in flexion. The medial compartment is also expected to have 1–2 mm more laxity while in flexion compared to when in extension (40). Furthermore, osteophytes significantly disrupt intraoperative joint balancing during TKA by acting as space-occupying lesions that alter ligament tension and joint contact mechanics. In a typical varus osteoarthritic knee, medial compartment osteophytes create apparent medial tightness by tenting the medial soft tissues, resulting in a falsely narrowed medial gap. Conversely, osteophytes on the lateral tibial plateau may exert the opposite effect depending on their size and interaction with surrounding structures. Large posterior femoral condylar osteophytes further contribute to imbalance by increasing tension in the posterior capsule, limiting terminal knee extension and promoting flexion contractures. These changes generate asymmetric medial–lateral gap measurements that do not reflect the native ligamentous state. Importantly, the biomechanical effects of osteophytes are highly variable and patient-specific, depending on osteophyte volume, location, and the manner in which surrounding ligaments and capsular structures wrap or articulate against them.

Because these effects cannot be reliably predicted or accounted for during the initial balancing stage, any attempt to adjust gaps prior to osteophyte removal and restoration of native bony anatomy is inherently unreliable. Any attempt to “balance” the knee at this stage is premature and may lead to inaccurate gap assessment, altered knee kinematics and distorted ligament isometry that ultimately results in a biomechanically imbalanced knee (43).

In KA, the goal is to restore the natural difference in symmetry and varus/valgus laxity between extension of 0° and flexion of 90° of the knees. Hence, regardless of how unbalanced the gaps may appear during the initial gap assessment, the senior surgeon proceeds with bone resection without making any adjustments to implant positioning, in adherence to the principles of kinematic alignment (15). This differs from MA, where the aim is to achieve gap-balancing and matching of the knee's laxity in extension and flexion, which can cause stiffness, abnormal kinematics and accelerated implant wear. Therefore, in KA, no soft tissue balancing or ligament manipulation are needed. Through the bone cuts alone, the knee will revert to its natural alignment and balance. KA without ligament release limits high compartment forces by restoring those of the native knee (44–46). Literature shows that there is no medial or lateral compartment overload in KA, even in patients with varus-valgus alignment that lie in the outlier range according to MA criteria (47).

## Performing and validating the bone cuts

The next step is to bring the Mako® robotic arm into the field and perform the bone cuts. The cuts are performed according to the pre-operative plan made. The femoral resections are performed first, followed by the tibial resection. Each cut is measured and verified using callipers to ensure accuracy before proceeding to the next step. The femur order of resection is as follows: distal femur, posterior chamfer, anterior femur, anterior chamfer and finally the posterior femur. All retractors should be positioned prior to the introduction of the robotic arm to aid exposure. This consists of a lateral collateral ligament retractor placed on the lateral aspect of the femur to protect the lateral structures and the patella tendon and a Z-retractor placed medially to protect the medial collateral ligament. With the AccuStop™ haptic technology, the saw is constrained to the virtual boundaries established by the pre-operative plan, and this is reflected on the screen. This results in less direct visualisation needed of the knee as the cut trajectories are guided by the robotic arm. Thus, skin and soft tissue can be preserved as less soft tissue damage from exposure of the bone is needed.

As mentioned, the senior author will still verify the bone cuts using “callipered” kinematic alignment technique described by Howell (16). The femoral and tibial bone resections are within ±0.5 mm of the target resection thickness. In a typical knee with varus osteoarthritis, the distal medial femur resection should measure 4.5 mm. This value is derived by subtracting 2 mm to account for the Mako® saw blade kerf from the implant thickness of 8.5 mm, followed by subtracting 2 mm to account for full articular cartilage loss (31, 32). For the distal lateral femur in a varus knee without cartilage loss, the expected resection thickness is 6.5 mm. This is calculated by subtracting 2 mm to account for the saw blade kerf from the femoral implant thickness of 8.5 mm, with no further adjustment required since there is no cartilage wear in this region. For the tibial resection, which corresponds to a tibial implant thickness of 9 mm, the expected calliper measurement is approximately 7 mm from the base of each tibial spine. This is calculated by subtracting 2 mm to account for the saw blade kerf from the implant thickness, ensuring accurate bone resection and proper restoration of the native joint line.

## Evaluation of soft tissue tension and implantation

**Figure 4:**
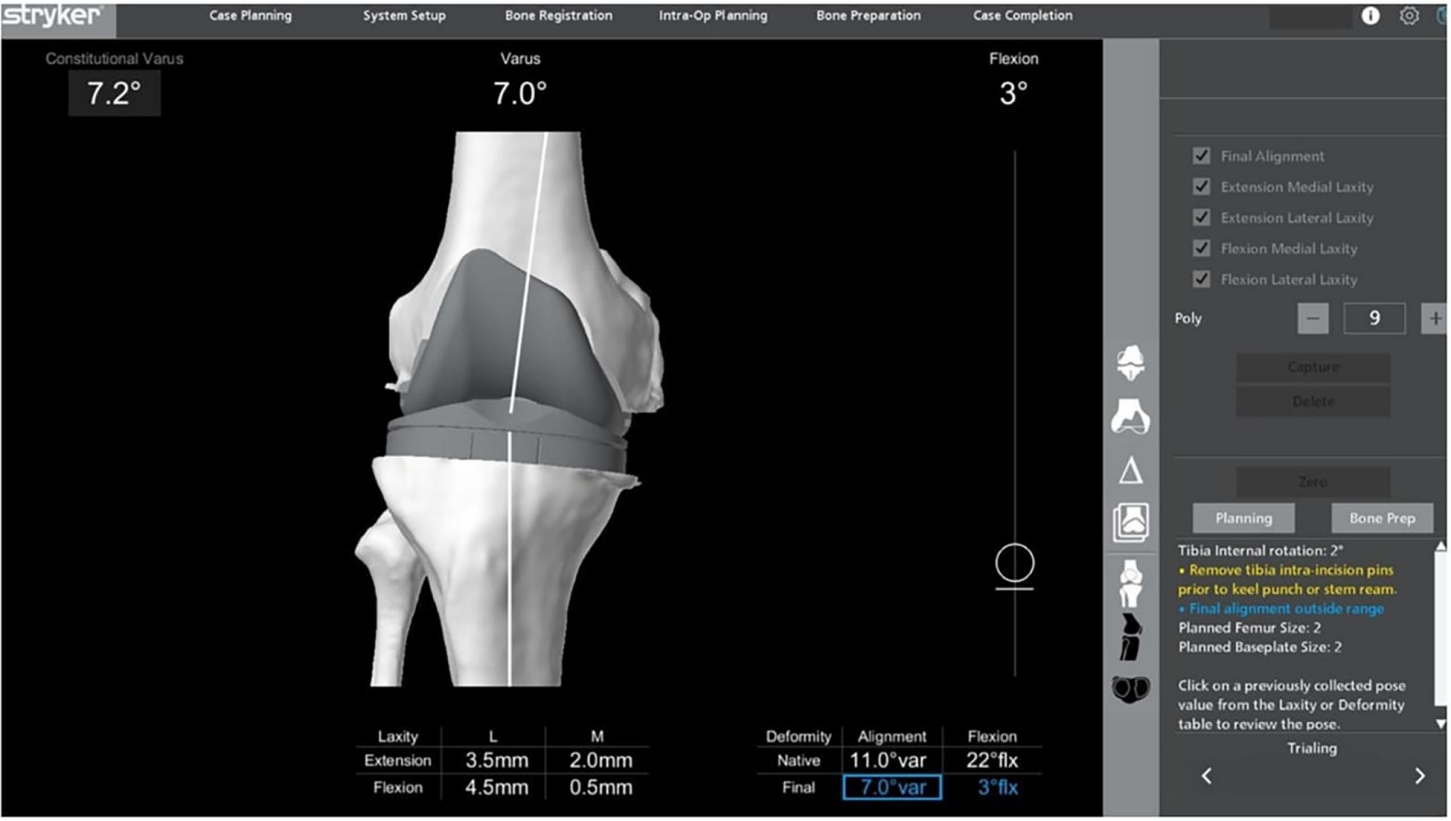
Final evaluation with trial implants. Alignment has corrected to 7° varus from 11° varus as initially planned. Medial and lateral laxities displayed are acceptable with the lateral compartment being more lax.

After the bony resections have been completed, the menisci and posterior osteophytes are removed. Implant trialling is then performed. Evaluation of the trial implants involves both clinical assessment and robotic measurements (Figure 4).

The first intraoperative check focuses on restoration of the patient's native limb alignment and elimination of varus–valgus laxity with the knee in full extension (0°). It is important to keep in mind that the aim in KA is to restore the knee back to its pre-arthritic alignment, which is usually not perfect neutral alignment (11–14). Thus, even if the knee is in varus or valgus alignment, the senior author is not concerned. At this stage, the surgeon performs a clinical assessment of knee stability, which is complemented by quantitative gap measurements provided by the Mako® system.

With the knee in 90° of flexion, the trial tibial insert should fit tightly in the medial compartment and more loosely in the lateral compartment, allowing the knee to pivot around the medial side. The medial compartment is designed to remain stable, with approximately 1–2 mm of residual laxity, while the lateral compartment is allowed greater laxity, often 1–5 mm more than the medial side. This recreates a trapezoidal flexion space that closely mimics the kinematics of the native knee. The tibia must be able to internally and externally rotate like the native knee in 90° of flexion. The knee is then brought into full extension, and varus–valgus laxity is assessed. The goal is a stable knee with symmetric medial and lateral gaps and no residual coronal plane laxity. On the Mako® system, the surgeon typically aims for extension gaps of less than 1 mm on both the medial and lateral sides.

**Figure 5:**
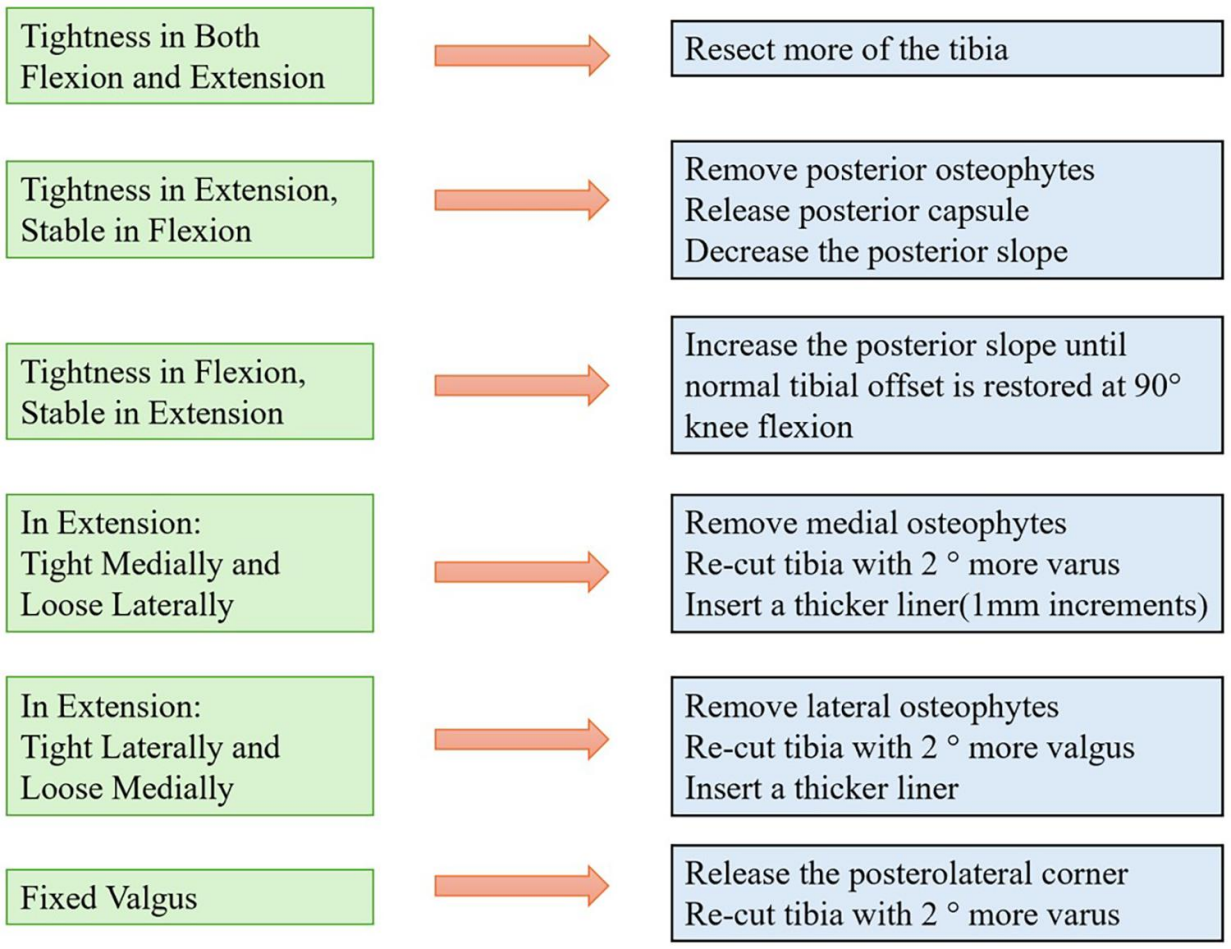
Robot-guided decision tree for addressing residual imbalance during kinematic alignment total knee arthroplasty. Intraoperative decisions are informed by quantitative gap measurements and alignment data provided by the Mako® system in conjunction with clinical assessment. Adjustments are performed in a stepwise manner, beginning with osteophyte removal, followed by selective posterior capsular release, and finally tibial-side modification if required.

As mentioned, the goal in KA is to restore the natural difference in symmetry and laxity observed between knee extension and flexion (15). In addition, collateral ligament laxity is not isometric through the arc of knee flexion (9). Thus, in addition to the numerical values seen on the Mako® System, clinical judgement is of utmost importance when assessing for acceptable laxities in the knee. Clinical validation is performed concurrently to confirm a medial pivoting knee pattern, absence of excessive tightness, and physiologic rotational freedom. At 90° of flexion, the knee should allow approximately 10° of internal and external rotation without lift-off of the trial liner, confirming appropriate soft-tissue balance and functional kinematics. If the knee is found to be imbalanced, adjustments are performed in a deliberate and sequential manner to preserve kinematic alignment principles (Figure 5).

Osteophyte removal is performed first, as osteophytes act as space-occupying lesions that produce misleading gap measurements. Removal of marginal and posterior osteophytes restores native bony contours and allows reassessment of true ligament behaviour.

Posterior capsular release is considered next, but only if a flexion contracture or extension tightness persists after osteophyte removal and restoration of planned bone resections. Posterior capsular tightness, often exacerbated by posterior condylar osteophytes, may limit terminal extension and falsely suggest imbalance.

If necessary, tibial adjustments are performed last once osteophyte-related and capsular constraints have been addressed. Any tibial modification is used to fine-tune residual imbalance while preserving femoral anatomy, joint line obliquity, and kinematic alignment principles. Femoral component position and ligament releases are intentionally avoided whenever possible, as altering femoral anatomy risks violating native ligament isometry and knee kinematics. Incremental adjustments are limited to ≤2° to allow controlled, reversible fine-tuning while preserving native joint line orientation, ligament isometry, and kinematic alignment principles. Although the Mako® system provides high execution accuracy, larger corrections are intentionally avoided to reduce the risk of overcorrection and unintended alteration of patient-specific knee kinematics.

**Figure 6:**
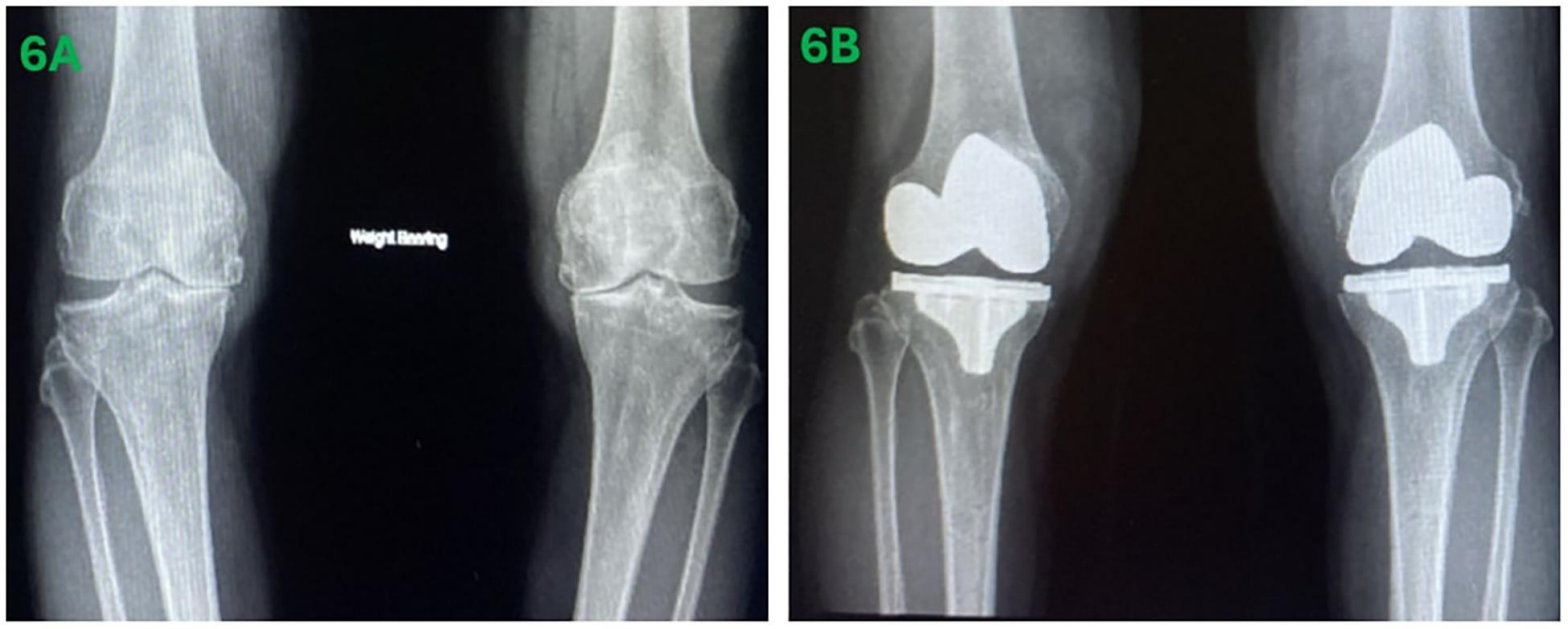
Pre-operative anteroposterior films of bilateral knees **(A)** and post-operative films of a patient that underwent Mako® assisted kinematically aligned total knee arthroplasty **(B)**.

With the added precision of the Mako® 2.0, the chance of needing to perform bone re-cuts to balance the knee is expected to be lower as compared to conventional calliper technique. Existing literature has shown that when compared with conventional TKA, Mako®-assisted TKA achieves more precise bone cuts and alignment (24–26). Radiographic films of a patient with severe valgus deformity that underwent Mako® assisted KA TKA for both knees at our institution are provided for illustration (Figure 6).

## Discussion

### Expected outcomes

Initially described by Insall et al., MA has been the widest used method for TKAs (3, 48). Although a mechanically aligned TKA improves function, literature has shown that there remains a high rate of dissatisfaction in patients, reaching almost 20% in some reports (5–7). KA was developed to improve knee function and pain control. As opposed to MA, which mainly focuses on “straightening” the knee in the coronal plane, KA aims to co-align the axes of the femoral and tibial components with the three axes of the native knee which takes into accounts the rotational and sagittal profiles of the knee (16).

There are several comparative studies that have reported functional outcome advantages of KA over MA (19, 22, 49, 50). Dossett et al., compared two-year outcomes between KA and MA TKAs and found that the KA group provided a better chance of a pain-free knee and significantly better range of motion (*p* = 0.002). Their KA group also had better Knee Society Score (KSS), Western Ontario and McMaster Universities Osteoarthritis Index (WOMAC) and Oxford Knee Score (OKS) results that were statistically significant (*p* = 0.005) (22). In Japan, Matsumoto et al., compared navigated KA vs. MA TKAs and reported significantly better knee flexion (*p* < 0.003) and KSS scores (*p* = 0.03) in the KA group (50). In a meta-analysis on KA vs. MA TKAs involving 1,112 cases, the KA group achieved better KSS, WOMAC and knee flexion results compared to the MA group (19).

With regards to implant longevity, it has been shown that achieving MA does not result in better survivorship. In a large clinical series, Abdel et al. compared 20-year implant survivorship rates between MA TKAs against TKAs that fell outside of ±3° relative to the mechanical axis and found that MA did not improve implant survival (51). There is a relative paucity on implant survivorship for KA TKAs, but the available literature reports good implant survivorship. Howell et al. reported a 16-year survivorship rate of 93% (23). Klasan et al. performed a large observational study on 20,512 cases of KA TKAs and reported a cumulative 7-year revision rate of 3%–3.1% (20).

Conventional TKAs can achieve a high degree of accuracy when performed by experienced surgeons. However, there have been multiple studies that have shown that robotic assisted TKAs can provide superior resection accuracy compared with conventional jig-based techniques (24–27). Beyond resection accuracy, robotic assistance offers several advantages that are particularly relevant to KA, including enhanced soft-tissue protection, reduced iatrogenic soft-tissue trauma, objective quantification of gap behaviour and ligament tension, and real-time intraoperative alignment verification. Prior work by Hampp et al. has demonstrated reduced soft-tissue damage with robotic assisted TKAs compared with manual techniques, likely attributable to enhanced preoperative planning and real-time intraoperative feedback (52). Additional studies have shown improved early functional recovery, reduced pain, faster rehabilitation, and decreased narcotic use following robot assisted TKAs (24, 25, 27).

There is limited literature on outcomes of specifically robotic assisted KA TKAs as well as direct comparison studies on robotic assisted KA vs. MA TKAs. This area will benefit from further research especially with the advancement of technology in the field of Orthopaedics. One of the major challenges in knee balancing, regardless of alignment philosophy, is the inherent subjectivity associated with manual force application during intraoperative gap assessment, which makes standardization and quantification difficult. The Robotic evaluation of articular laxity (REAL) classification is an example of an attempt to address this challenge; however, this was developed using the ROSA robotic system within a functional alignment strategy (53). Further research on the development and validation of a laxity or balancing classification system through the Mako® system could be beneficial.

## Conclusion

To perform a KA TKA effectively, accurate bone resections, particularly the femur aspect, are required to ensure that the pre-arthritic alignment and joint line are restored. The Mako® system provides accurate and precise assistance in performing a KA TKA through the combination of meticulous 3D CT-based pre-operative planning and AccuStop™ haptic technology. There is a paucity in studies that describes the surgical technique of performing a KA TKA while using Mako® Total Knee 2.0 system. This article describes a practical and efficient workflow that integrates kinematic alignment principles with the precision, reproducibility, and intraoperative feedback provided by robotic assistance.

## Data Availability

The raw data supporting the conclusions of this article will be made available by the authors, on reasonable request.
